# HOUSING CHARACTERISTICS, NEIGHBORHOOD ENVIRONMENTS, AND SELF-RATED MENTAL HEALTH AMONG OLDER CANADIANS

**DOI:** 10.1093/geroni/igac059.2308

**Published:** 2022-12-20

**Authors:** Ethan Siu Leung Cheung, Ada Mui

**Affiliations:** Columbia University, New York, New York, United States; COLUMBIA UNIVERSITY, New York City, New York, United States

## Abstract

Previous studies have established a clear association between the surrounding environment and mental health. Whereas most literature has focused on neighborhood environment, very few studies have examined the role of housing characteristics in self-rated mental health (SRMH). Using data from the 2018 Canadian Housing Survey, this study investigated the relationships between housing characteristics, neighborhood environment, and SRMH among older Canadians and whether the relationships varied by education and gender. Using a sample of 21,725 Canadians, SRMH was measured by older adults’ self-evaluation of mental health on a 5-point scale. We categorized education into three groups: high school or less, some college, and university or beyond. Hierarchical linear regressions showed that men and women with high school education and women with some college educations were more likely to report worse SRMH when living in low-income housing. Reporting a home maintenance need was a unique risk factor of SRMH for men with a university education, whereas living in uninhabitable conditions uniquely predicted better SRMH for men with some college education. Regarding neighborhood environment, safer community was a protective factor of SRMH for women with university education only. Sense of belonging was positively associated with SRMH for all subgroups, except for men with a university education. Expressing a need for community service was significantly associated with lower SRMH for women who completed a high school education or some college. Findings of this study shed light on the diverse need for environmental improvement and maintenance programs to improve SRMH among older Canadians.

